# Supplementary material for: Analysis of molecular and cellular bases of honey bee mushroom body development
Source: Sci Rep. 2025 Jul 1;15:21462. doi: 10.1038/s41598-025-06268-3 (PMC12215803; doi:10.1038/s41598-025-06268-3)
Supplement: Supplementary file 1 — Supplementary Material 1 [file 41598_2025_6268_MOESM1_ESM.pdf]

Supplementary Information for

Analysis of molecular and cellular bases of honey bee mushroom development

Shuichi Kamata[1], Takeo Kubo[1], Hiroki Kohno[1]*

[1]Department of Biological Sciences, Graduate School of Science, The University of Tokyo, Bunkyo-ku, Tokyo 113-0033, Japan.

* Corresponding author

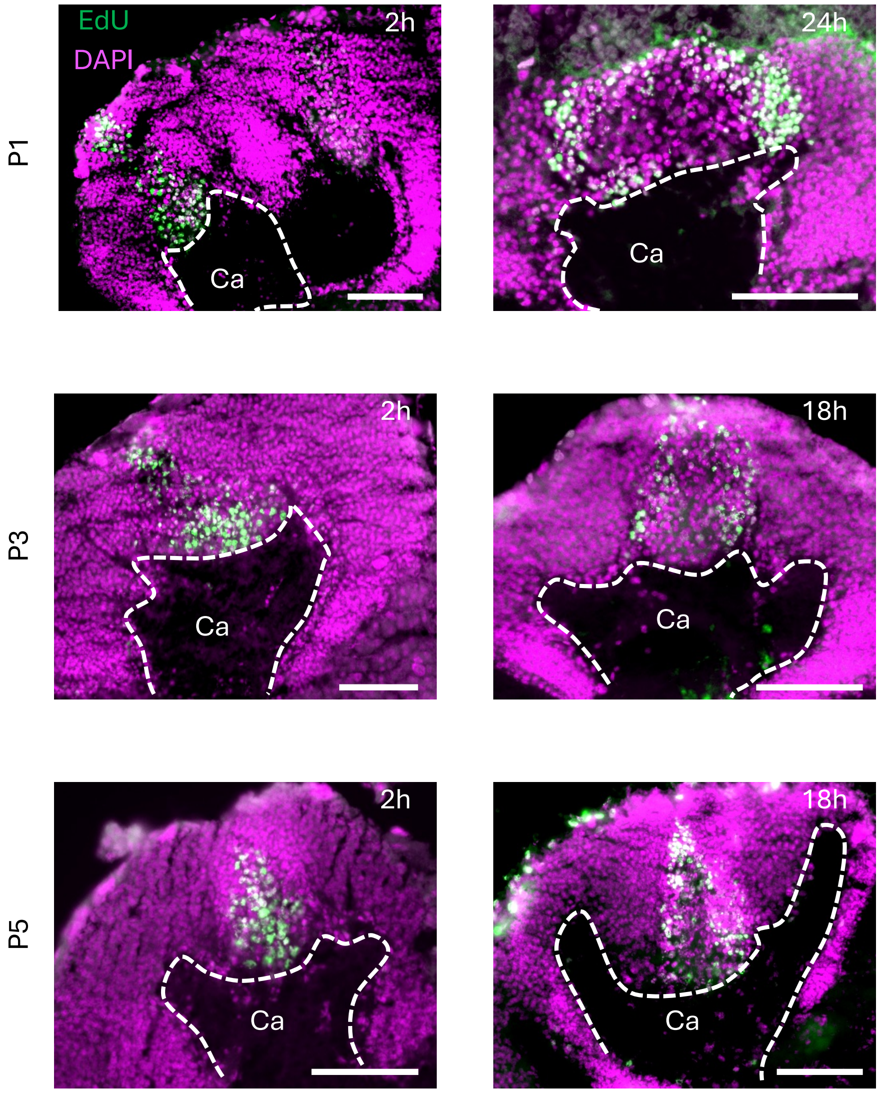

Figure S1 Changes in signal distribution of EdU injected into the pupal MBs.
Marged images for EdU and DAPI signals in the MBs of pupae at P1, P3 and P5. The time shown in the upper right of each panel indicates the time after EdU injection. Ca, calyx. Scale bar; 100 μm.

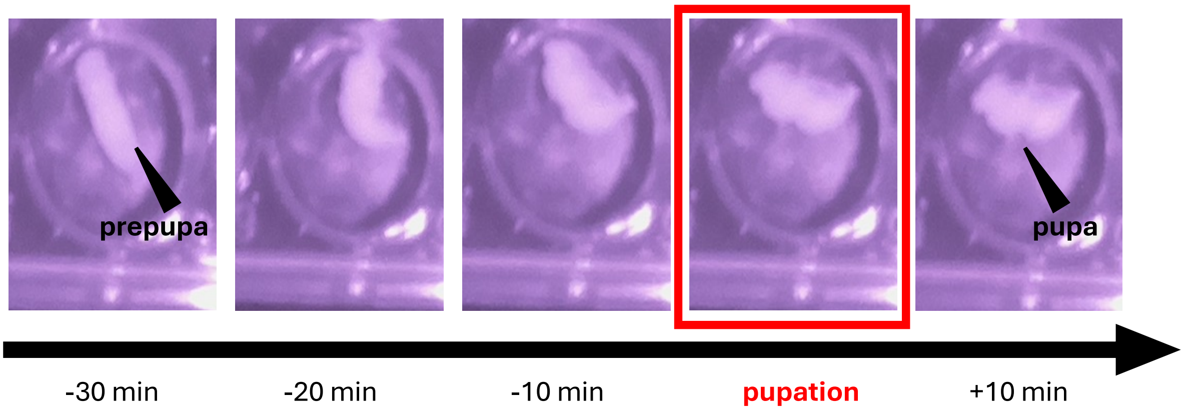

Figure S2 Time-lapse imaging of pupation in the artificial rearing condition
Time-lapse images (10 min increment from left to right) of an individual from prepupa to pupa under artificial rearing conditions are shown. '−30 min' indicates 30 min before pupation, and '+10 min' indicates 10 min after pupation. The abdomen begins to move immediately before pupation and ceases to move after pupation.

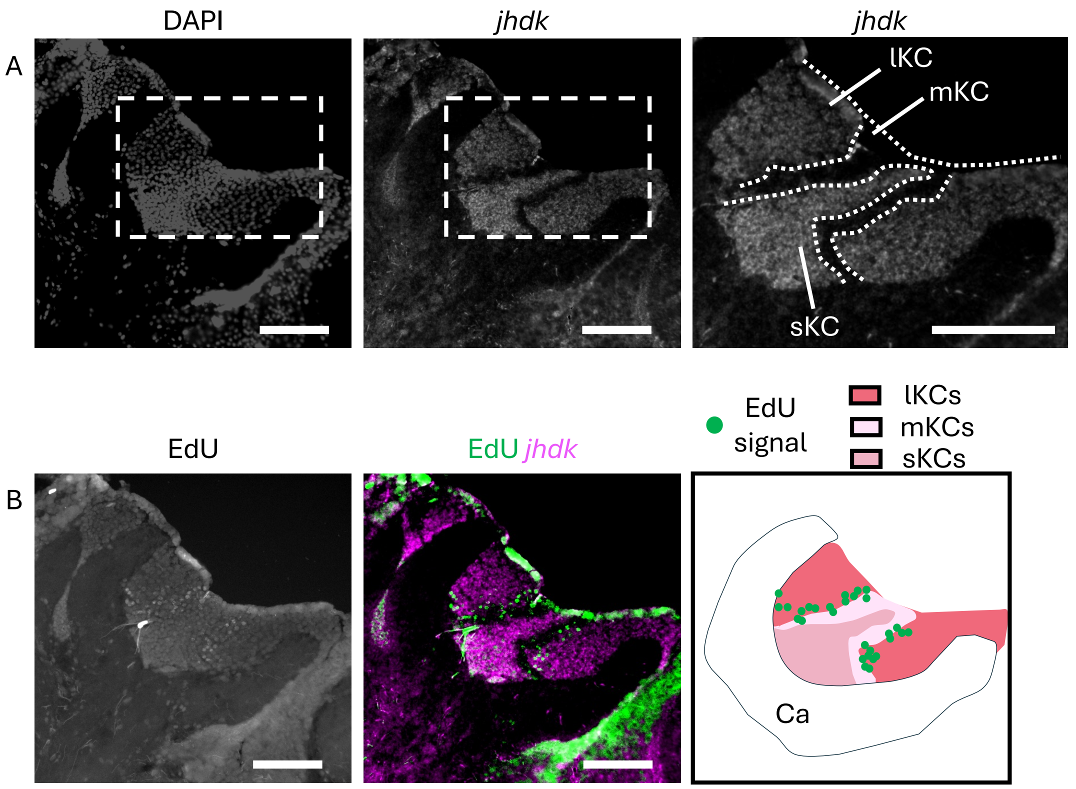

Figure S3 Identification of KC subtype in which EdU signals were detected in the adult MBs
(A)Discrimination of KC subtypes based on *jhdk* ISH and DAPI signals in the adult MBs. From left to right, DAPI signals, *jhdk* FISH signals, and magnified view of the *jhdk* FISH signals in the white dashed box in the middle panel are shown. Dashed lines in the right panel indicate boundaries of the lKCs, mKCs and sKCs. (B) Distribution of EdU signals in the adult MB of the individual injected with EdU at 24 hap. From left to right, EdU signals, merged signals of EdU and *jhdk*, and schematic diagrams of EdU signals and each KC subtype are shown. Ca, calyx. Scale bar; 100 µm.

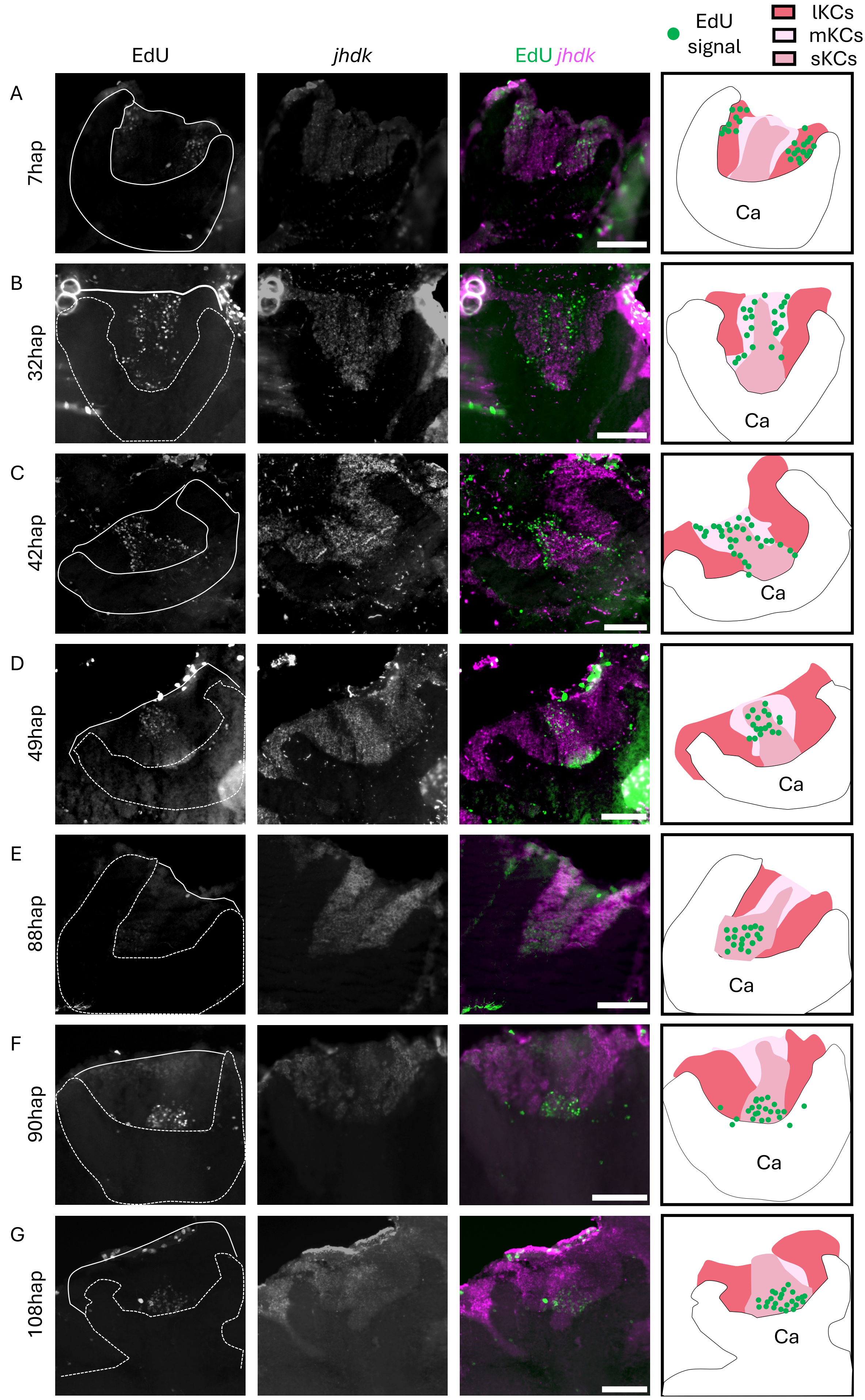

Figure S4 Identification of the pupal stage when each subtype is produced from neuroblasts
Distribution of EdU signals in the brains of adult individuals injected with EdU at various pupal stages: 7 hap (A), 32 hap (B), 42 hap (C), 49 hap (D), 88 hap (E), 90 hap (F), and 108 hap (G). From left to right, EdU signals, *jhdk* FISH signals, merged signals, and schematic diagrams of EdU signals and each subtype are shown. Ca, calyx. Scale bar; 100 µm.

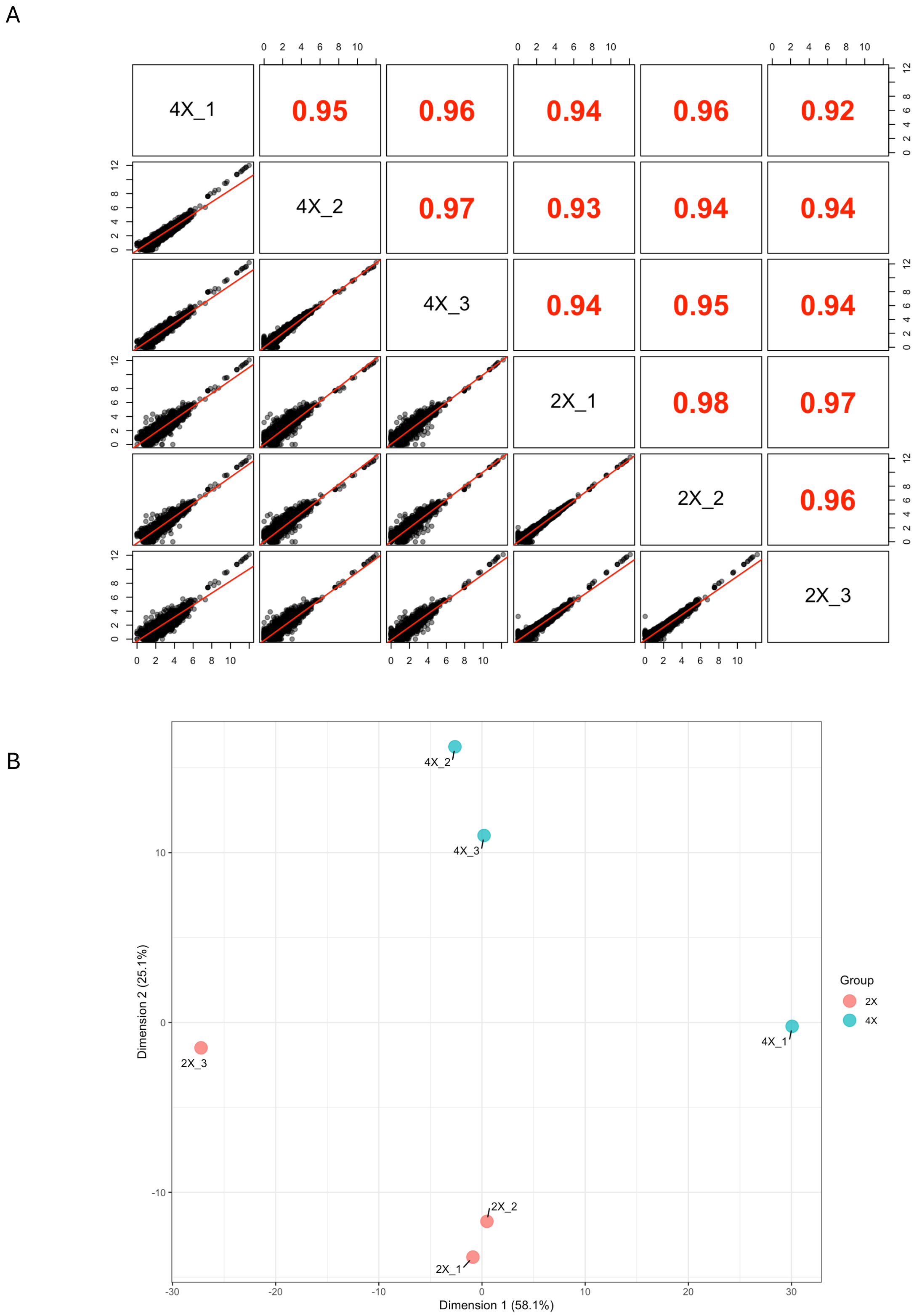

Figure S5 Validation of RNA-seq analysis
(A) Pearson’s correlation coefficient between each sample calculated using normalized expression levels. (B) Two-dimensional map of Classical Multidimensional Scaling (CMDS). Red and blue dots indicate biological replicates for 2X or 4X fractions, respectively. The horizontal and vertical axis represents the first or second principal component, respectively.

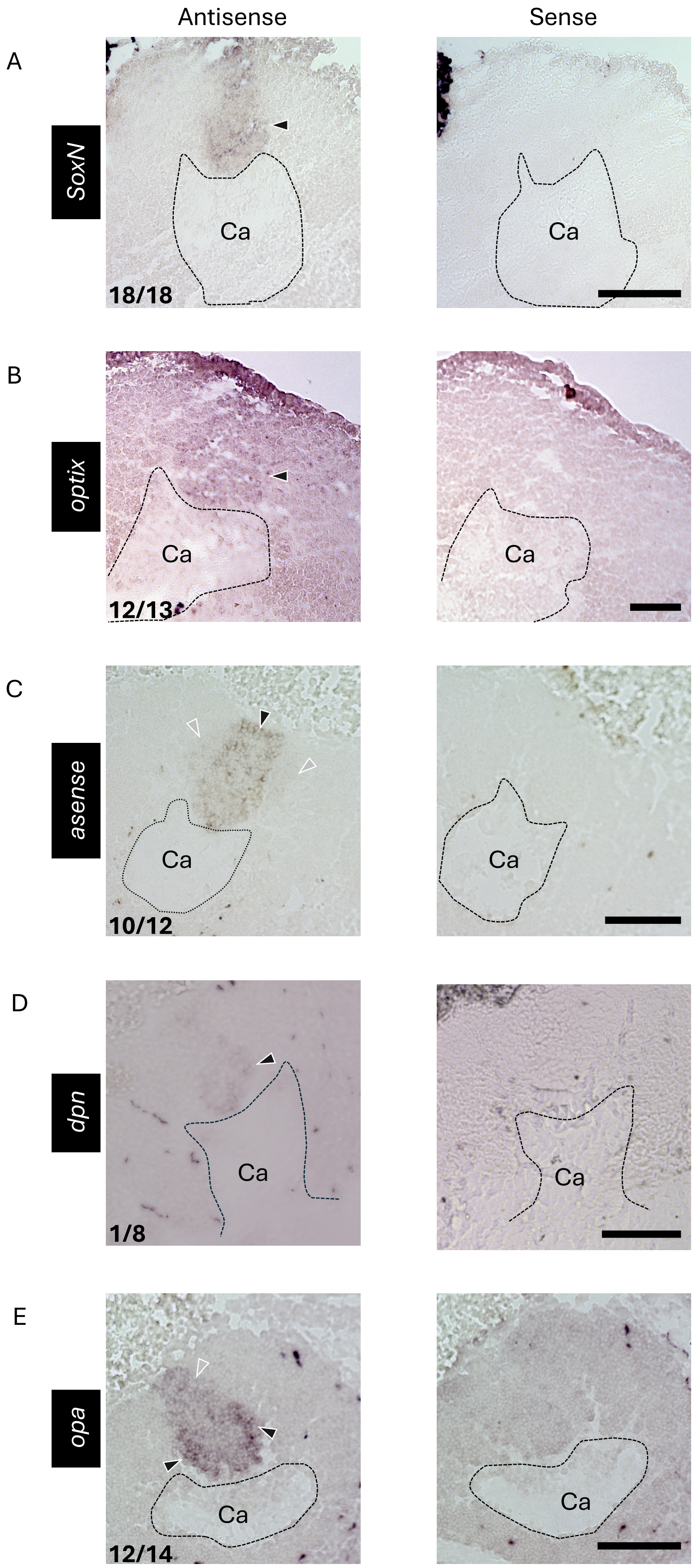

Figure S6 Antisense probe-specific ISH signals of the genes suggested to be expressed in the proliferating cells or immature KCs.

ISH results of *SoxN* (A), *optix* (B), *asense* (C), *dpn* (D), and *opa* (E) in the MBs of pupae with proliferating MB cells using antisense (left) and sense (right) probes. Black and grey arrows indicate strong and weak ISH signals, respectively, which were specifically detected using antisense probes. The fractions at the bottom corner indicate the proportion of pupal MBs with antisense probe-specific signals among the all MBs analyzed. Ca, a calyx in the MB. Scale bar; 100 µm.
